# Supplementary material for: Frequency and determinants of misuse of augmentation of labor in France: A population-based study
Source: PLoS One. 2021 Feb 9;16(2):e0246729. doi: 10.1371/journal.pone.0246729 (PMC7872232; doi:10.1371/journal.pone.0246729)
Supplement: S1 Table — AL = Augmentation of Labor; NA = not applicable. (PDF) [file pone.0246729.s001.pdf]

S1 Table. Rate and characteristics of rupture of the membranes and oxytocin infusion in the groups of augmentation of labor

	Standard use of AL N= 3225	Misuse of AL N= 1524
Women with intact membranes at admission, n(%)	2983 (92.5)	1462 (95.9)
Women receiving artificial ROM if membranes were intact at admission, n(%)	1939 (65.0)	1131 (77.4)
Duration between admission and artificial ROM when performed, hours, median ± SD	2.2 ± 1.7	1.0 ± 1.4
Women with artificial ROM <1 hour after admission if membranes intact, n(%)	*NA*	591 (40.4)
Women with oxytocin infusion during labor, n(%)	2112 (65.5)	1178 (77.3)
Duration between admission and oxytocin infusion, hours, median ± SD	3.2 ± 3.0	1.5 ±1 .8
Women with oxytocin infusion <1 hour after admission, n(%)	*NA*	410 (26.9)
Women with oxytocin infusion if membranes intact on admission, n(%)	1870 (62.7)	1116 (76.3)
Duration between ROM and oxytocin infusion if ROM during labor, median ± SD	3.0 ± 4.2	0.6 ± 1.8
Women with duration between ROM and oxytocin infusion <1 hour, n(%)	*NA*	857 (56.2)

AL= Augmentation of Labor
NA= not applicable
